# Exploring Ant‐Mollusk Interactions: Insights From Southern Spain

**DOI:** 10.1002/ece3.71326

**Published:** 2025-05-07

**Authors:** Jairo Robla, Omar Sánchez, Miguel Ángel Gómez‐Serrano, J. Manuel Vidal‐Cordero

**Affiliations:** ^1^ Estación Biológica de Doñana Consejo Superior de Investigaciones Científicas Sevilla Spain; ^2^ Department of Organisms and Systems Biology (Zoology) University of Oviedo Oviedo Spain; ^3^ Cavanilles Institute of Biodiversity and Evolutionary Biology University of Valencia Valencia Spain

**Keywords:** *Cecilioides acicula*, Formicidae interactions, *Granopupa granum*, hoarding behavior, *Messor* ants, myrmecophily, snail shells

## Abstract

Ants occupy a great variety of habitats, perform essential ecological roles, and interact with a wide variety of other organisms. However, the interaction between ants and mollusks is a lesser‐explored relationship that can be categorized into (a) ant predation on mollusks, (b) shell collection as hoarding behavior, (c) the use of shells for nesting, and (d) myrmecophilic relationships. This study reports new data about several interactions from accidental field observations, a quantitative analysis of the snail shells found in 16 *Messor* ant nest cleanings, and a qualitative analysis of 51 additional nests of different species. We found 1127 snail shells from 20 species, most of them belonging to juveniles of the Geomitridae and Helicidae families. Notably, *Granopupa granum* was the only species found alive in the collected material. Furthermore, in our qualitative assessment, we found 86.8% of the analyzed nests with shell remains in the nest cleanings of at least nine ant species. Additional observations revealed ants transporting both empty shells and live snails to the nest, some living snail species around the nest entries, and additional interactions. Our results may support cases of (a) predation of snails of certain species by ants, as many shells were found with perforations compatible with ant attacks and we have recorded direct predation, (b) the collection of empty shells to gather the body remains of snails as a trophic resource or for other purposes, and (c) the potential existence of more myrmecophilous snail species than currently known, capable of living in ant nests without being attacked, like *Cecilioides acicula* , *Ferrussacia folliculum*, or *G. granum*. Although more studies are necessary to understand the intriguing relationship between ants and snails, the study of ant nest wastes can also become a valuable tool for detecting rare native micromollusc, as well as invasive, non‐native, and aquatic species.

## Graphical abstract

This study reports new data about ant‐mollusk interactions from accidental field observations, a quantitative analysis of the snail shells found in 16 *Messor* ant nest cleanings, and a qualitative analysis of 51 additional nests of different species.

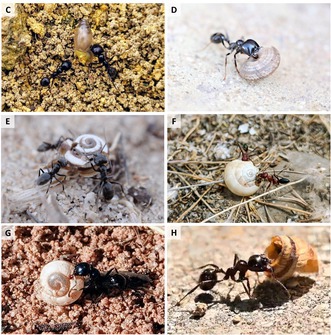

## 1 Introduction

Ants, with almost 14,300 known species (Bolton 2024), occupy a wide range of habitats and play crucial ecological roles such as seed dispersal, organic matter decomposition, and even pollination, interacting with numerous organisms in their habitats (Del Toro et al. 2012; Schultheiss et al. 2022). Furthermore, ants maintain a surprising diversity of interactions with other organisms, forming complex ecological networks ranging from mutualistic relationships to parasitic, commensalism, and predatory behavior (Wasmann 1894; Kistner 1982; Schmid‐Hempel 1998). While some associated groups, such as certain Coleoptera, Lepidoptera, Zygentoma, Acari, and specific families of Hymenoptera or Diptera, have been extensively studied (Hölldobler and Kwapich 2022), there are numerous organisms associated with ants whose biology remains poorly understood, like myriapods (Stoev and Lapeva‐Gjonova 2005) and terrestrial isopods (Oxman et al. 2024). Among these, mollusks represent a less explored interaction group that could be classified in different categories, offering an opportunity to broaden our understanding of the ecological dynamics between ants and neglected taxa (Vaisman and Mienis 2011).

Firstly, the most documented specific relationship in the literature, albeit often in a very scattered manner, is predation by ants. Mollusks form a specialist diet for tropical cryptic ants of the genus *Basiceros* Schulz, 1906 [i.e., *B. singularis* (Smith, 1858), *B. conjugans* Brown, 1974 and *B. manni* (Brown and Kempf 1960)] (Longino 1999; Probst 2015; Probst et al. 2019). Snails are also found in the more generalized diets of harvester ants of the genus *Messor* Linnaeus, 1767, specifically in species such as *M. capitatus* (Latreille, 1798) and *M. bouvieri* Bondroit, 1918 (Cerdá and Retana 1994), *M. ebeninus* Santschi, 1927 (Vaisman and Mienis 2011), and *M. wasmanni* Krausse, 1910 (Traxler 2016). Páll‐Gergely and Sólymos (2009) mentioned that snails of the genus *Monacha* Fitzinger, 1833, and juvenile *Helix* individuals collected by *Messor oertzeni* Forel, 1910, and *M. caducus* (Victor, 1839) species might be part of their diet, a possibility also noted by Bertrand (2010) for *M. bouvieri* . However, mollusks and other invertebrates are rarely collected in harvester ants compared to seeds and plant remains (Cerdá and Retana 1994; Traxler 2016). This predation relationship has been reported to endanger native and/or endemic snail species due to exotic and invasive ant species such as *Solenopsis invicta* Buren, 1972 (Forys et al. 2001), and *Pheidole megacephala* (Fabricius, 1793) (Uchida et al. 2016). Conversely, fire ant species of the genus *Solenopsis* (Westwood, 1840) (i.e., *S. geminata* (Fabricius, 1804) and *S. invicta* ) have been used as biocontrol tools for populations of invasive mollusk species, such as the apple snails *Pomacea paludosa* (Say, 1829) (Tennant and Porter 1991; Stevens et al. 1999) and *P. canaliculata* (Lamarck, 1828) (Yusa 2001). Moreover, ant predation on mollusks has also been reported as part of the diet of a variety of ant species (e.g., Kugler and Hincapié 1983; Dejean et al. 1993; Schatz et al. 1997; Fourcassié and Oliveira 2002; Araújo and Rodrigues 2006; Tan et al. 2019; Fonseca and Sant'Anna 2020; Carter et al. 2021).

A second type of interaction with mollusks involves ant species that exhibit hoarding or harvester behavior, collecting various small objects near their nests. Among these objects are mollusk shells, which may be mistakenly perceived as food resources (e.g., seeds) leading to their accumulation near the nest entrance (Forel 1874; Donisthorpe 1915; Weber 1941; Butot 1952; Urbański 1965; Mienis 1974; Seidl 1987; Páll‐Gergely and Sólymos 2009; Jahyny 2010; Vaisman and Mienis 2011). However, these shells may be used for other purposes already noted by other authors, such as modifying the nest internal or external temperature (Smith and Tschinkel 2007), obtaining protection from erosion and perturbation from water (Laundré 1990) and wind (Whitford 2003), or to mark their territory or nest (Gordon 1984). Furthermore, there are another interaction in which ants use snail shells for nesting, primarily cryptic ants species of the genus *Temnothorax* Mayr, 1861 (e.g., Bondroit 1911; Headley 1943; Bogusch et al. 2019; Tluste and Birkhofer 2021) or *Thaumatomyrmex* Mayr, 1887 (Jahyny 2010), among a great variety of other genera (see Emery 1894; Mukerjee and Ribeiro 1925; Brown and Kempf 1960; Weber 1969; Jahyny et al. 2003; Kikuchi and Tsuji 2005; Fokuhl et al. 2007; Jahyny 2010; Neckheim and Boer 2019).

A third type of interaction is myrmecophily, where mollusks live inside ant nests without being attacked. Heynemann (1868) first mentioned this with the description of a slug, *Veronicella myrmecophila* (now a junior synonym of *Pseudoveronicella liberiana* (A. Gould, 1850)), living with “big ants” but without additional comments. Then, Verdcourt (1957, 2002) described *Curvella myrmecophila* Verdcourt 2002, living inside ant nests and noting that juveniles are transported by ants. At the same time, Janssen and Witte (2002) described *Allopeas myrmekophilos* R. Janssen, 2002, which lives with the legionary ant *Leptogenys distinguenda* Jerdon, 1851. During the sedentary phase of the ant colony, the snail feeds on prey collected by the ants and lives inside their nest, and during the nomadic phase, the snails are transported by the ants (Witte et al. 2002). Three years later, Eguchi et al. (2005) reported four different snail species inside Ponerinae ant nests that likely scavenge on animal matter in garbage dumps and can be transported by the ants. Finally, Dias‐Soares et al. (2023) provided a detailed description of the interactions of eight gastropod species inside the nests of *Neoponera verenae* Forel, 1922, including ethological remarks. However, there are additional records of several living snail species in nests of different ants, but without information about the interaction and with doubts about whether these species can be classified as myrmecophiles (Bertrand 2010; Peixoto et al. 2010; Castaño‐Meneses et al. 2015, 2019; Araujo et al. 2019; Neckheim and Boer 2019). Although more studies are necessary to understand the nature of these relationships, snails likely establish a commensal relationship with their ant hosts (Witte et al. 2002; Parmentier 2021; Dias‐Soares et al. 2023). Another notable interaction is the first known case of kleptotrophobiosis. The snail *Euglandina aurantiaca* (Angas, 1879) establishes trophobiosis relationships with lantern bugs of the family Fulgoridae. Ants of the genus *Camponotus* Latreille, 1802, unable to obtain honeydew directly from the lantern bugs due to their small size, climb onto the snails' heads and lick the honeydew deposited on their bodies (Naskrecki and Nishida 2007).

Given the paucity of information on the relationship between ants and mollusks, the main goals of this work are to (a) report several cases of this overlooked relationship between ants and mollusks in the Iberian Peninsula, supported by accidental and systematic sampling and graphical material, and (b) discuss and provide hypotheses about the role of these interactions.

## 2 Material and Methods

**FIGURE 1:**
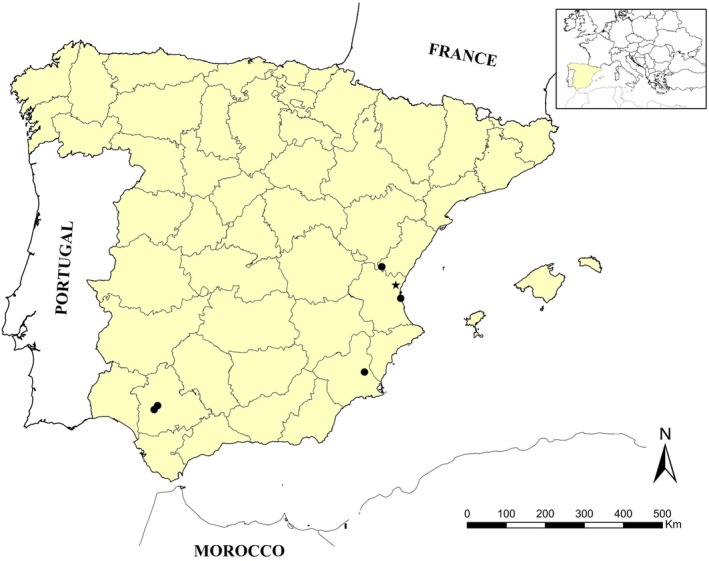
Location of the different observations: Systematic sampling (star) and accidental observations (circle) in Spain (yellow) in the European context (small map).

**FIGURE 2:**
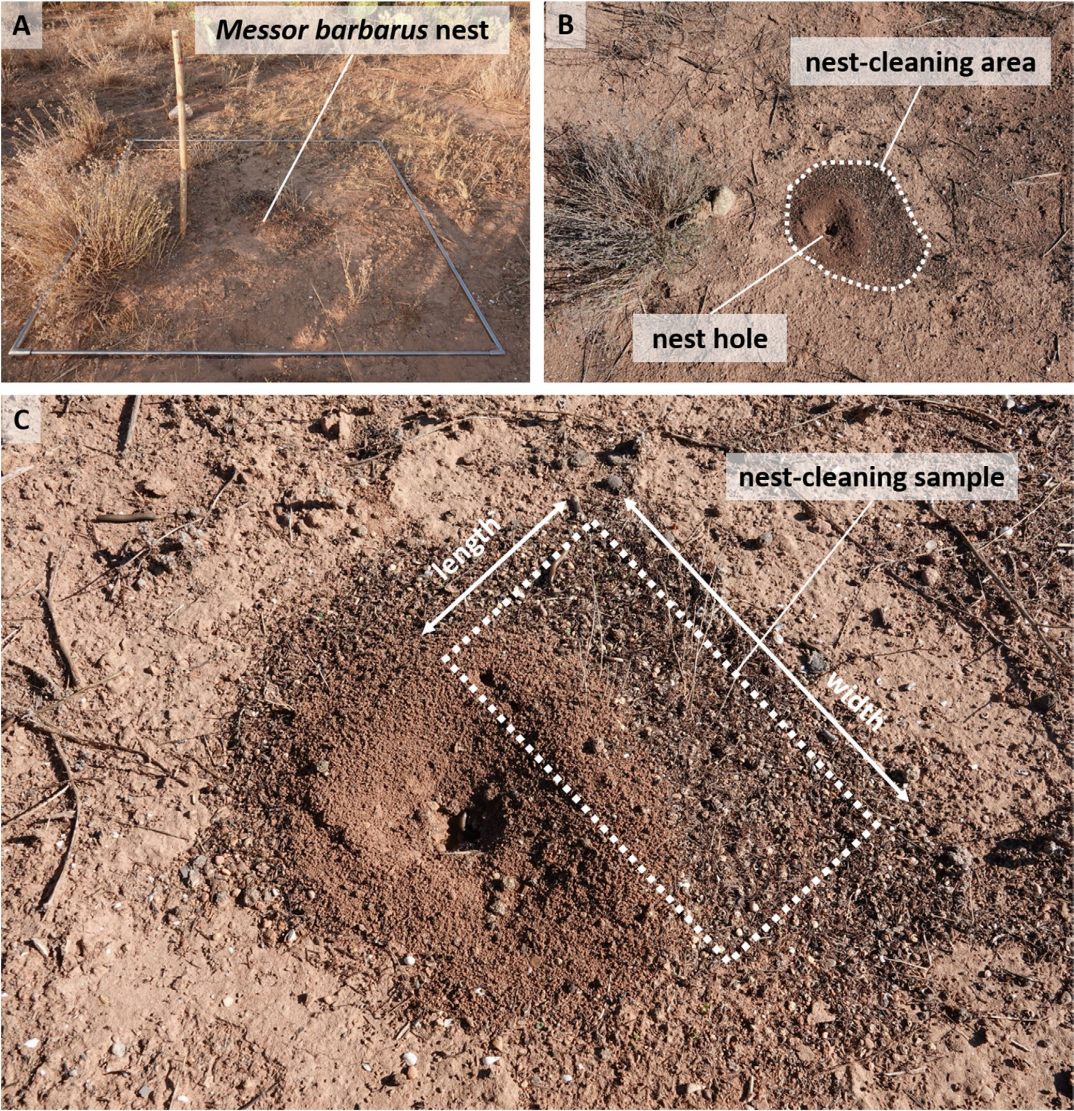
(A) Example of the external structure of a *Messor barbarus* nest (with the sticks that marked each nest) showing the nest cleaning accumulation area with respect to the nest entrance hole (B) and the area where the debris sample was collected (C).

Recorded interactions between mollusks and ants come from two different sources: (a) fortuitous encounters in the field, sampling for other purposes and observations kindly provided by other collaborators, and (b) an extensive sampling in ant nest cleanings (or refuse area, the area where the ants deposit wastes and debris from their nest). All these observations came from several parts of the Iberian Peninsula (Figure 1). The extensive sampling (b) study area was located in a Mediterranean coastal plain (Valencia) (Figure 1), crossed by a dry watercourse in an agricultural environment with small fragments of natural vegetation, dominated by scrub and forests of Aleppo pine *Pinus halepensis* Mill. The ant nests were found mainly in the watercourse bed (most of them less than 25 m far from the watercourse; middle point: 39.583135, −0.417946) or in the lateral sediment banks (two points: 39.550083, −0.427610; and 39.552756, −0.382881). The vegetation was dominated by gramineous grasslands and shrubs. In general, it is a waterless river, but in some areas, waterlogging occurs where marsh communities develop. During a study on seed mobilization by ants in this Mediterranean area, initiated in February 2022, a total of 67 active nests were inventoried, marked for identification (with nailed stakes and sticks less than 25 cm from the nest entry) (Figure 2A) and monitored over time. Fifty‐nine (88.1%) of the nests were in the dry watercourse, and the remaining 8 (11.9%) in two fragments of scrub habitat located 0.9 and 3.8 km from the water line. On the same day they were discovered, or on subsequent days if they were inactive, we collected a sample of the ants returning to each nest for accurate species identification. A list of all nests can be consulted in Appendix 1. The nests were photographed from multiple positions and distances (zenithal shot that covers the area of the nest entry and the refuse area, as well as several side shots from different directions) for later identification of the contents of the ant nest cleanings. The nest cleaning debris sample was collected from the fresh accumulation area near the nest entrance hole (see Figure 2) for later analysis to identify seeds and record their abundance. When the processing of the nest cleaning samples was advanced, during successive visits to the study area, several cases of ants transporting snails to the nests were recorded. These unexpected findings prompted the design of a specific methodology to assess the prevalence of these snail shells carrying among the different ant species.

Since most of the nest cleaning samples had already been processed and discarded, most of the nests were revisited to collect new samples. Nevertheless, some of the nests were inactive and had not incorporated new material to the outside of the nest and others had been disturbed by a water flood or by the widening works of a road annexed to the study area. Because of these limitations, we were only able to analyze a sample of nest cleaning (remains located outside the nest, 0 to 30 cm from the nest entry) (Figure 2B) from 16 nests of *Messor* ants to identify the snail species and assess their density from the volume of debris collected. Since the amount of debris present in each of the nests was not homogeneous, the volume of remains was used as a standardization measure, recording for it the length, width, and depth of the sampled nest cleaning area (Figure 2C). From these dimensions, we calculated the nest cleaning volume collected in cubic centimeters, which ranged from 25 to 400 cm^3^ (0.025–04 dm^3^). Samples were stored dry in hermetic individualized plastic bags until analysis. The remains of the 16 collected *Messor* nest cleaning samples were carefully examined to separate the shell of the snails present in the nests. All shells found were stored in hermetic plastic bags at room temperature until they were analyzed to identify the species involved and their abundance in the sample. From this count, the density of each species per unit volume was obtained. The taxonomic identification of ants and mollusks was carried out following the keys and the information provided in Arcos and García (2023) for ants and MolluscaBase (2024) for mollusks, under a Nikon SMZ dissecting stereomicroscope. Photographs of the snails were taken with a Canon EOS 1200D Digital SLR Camera with EF‐S 18–55 mm f/3.5–5.6 III lens and photomicrographs were taken with a Nikon Digital Sight DS‐L1 camera mounted on the previous stereomicroscope. In addition, we photographed and recorded videos of snail carrying by ants with a SONY DSC‐RX10M4 camera in our study area. In these accidental cases, ants were followed to their respective nests to check whether they introduced the snails inside them. In one of the observed events, the snail was collected before it was introduced into the nest to identify the species and examine the contents inside the shell (i.e., body remains, living specimen, and empty shell). The extra observations provided were carried out and kindly provided by different collaborators using their personal photograph equipment or smartphones in different locations of southwestern Spain (Figure 1).

In addition to this quantitative and descriptive approach, we conducted a qualitative assessment of the presence of snails in the nest cleaning for the whole sample of nests (*N* = 67). Quantitative analysis could only be performed in nests of *Messor* ants (*N* = 16), but since we had photographs of all the nests, the aim was to detect and highlight the presence of snail shells also in the remaining nests (several extra *Messor nests* and non‐*Messor* species nests; *N* = 51). To this end, we used previous photos of the nests to identify the presence or absence of snails on the surface of the nest cleanings and, if applicable, the species involved. To do this, the various photographs of each nest were opened in an image editor, and zoomed in. We attempted to locate and identify which snail species were present in the ant cleanings. Since many species are not discernible in the photos due to their small size, could be easily misperceived with other remains, or do not have clearly visible diagnostic characters for precise identification, we only considered the presence of those taxa that could be clearly identified with the photographic material and only to know whether shells appear in the nest cleanings of the remaining nests. Maps were generated with ArcMap 10.8.1, and graphs were made with Microsoft Excel 365.

## 3 Results

### 3.1 Ant Nest Cleanings

**FIGURE 3:**
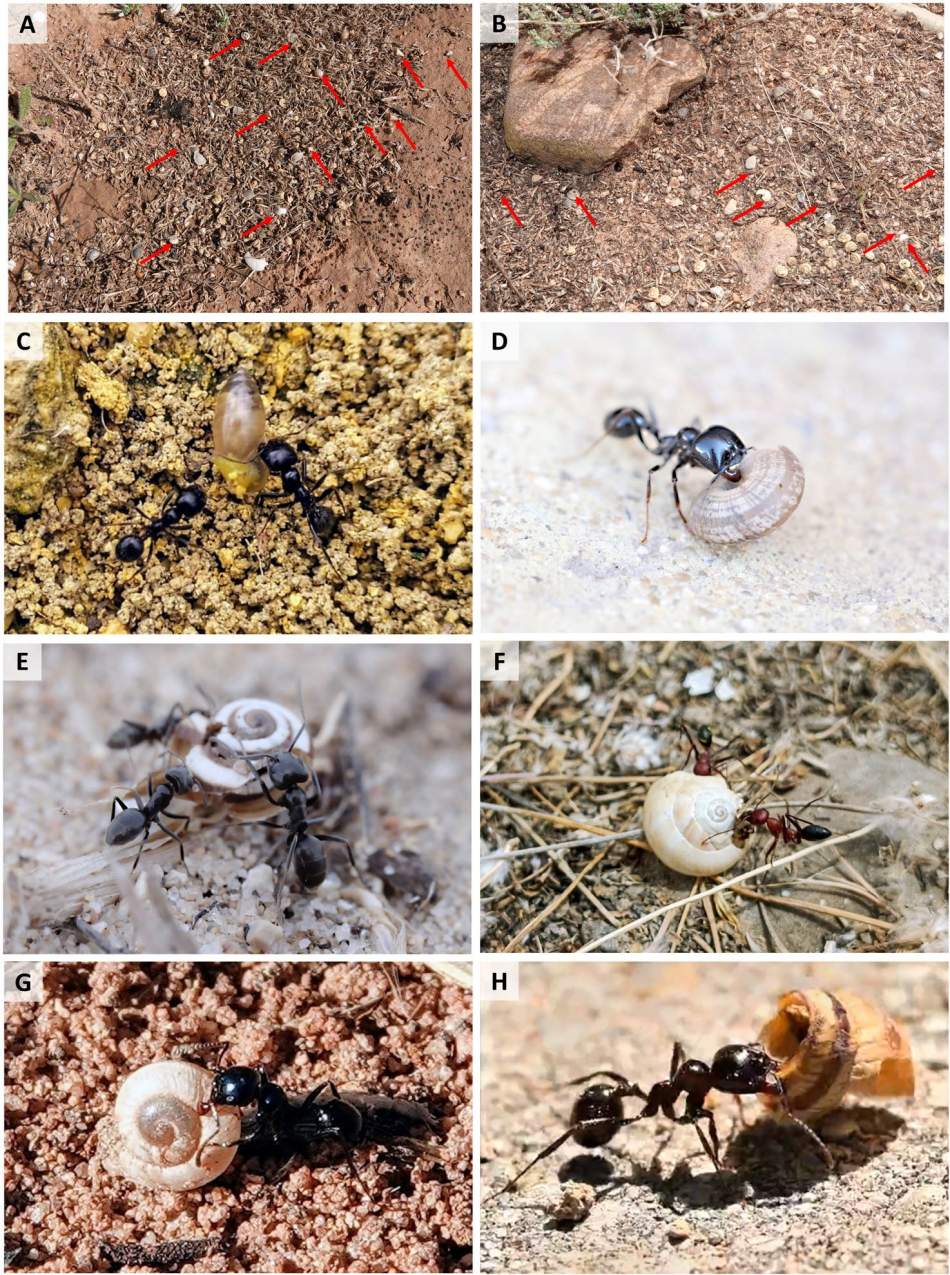
Ant nest cleanings of (A) *Messor barbarus* and (B) *Messor bouvieri* , where remains or whole snail shells can be observed (red arrows). Several ant species carrying shells. (C) *Messor barbarus* carrying an alive specimen of *Ferrusacia folliculum* (Photo credit: Jairo Robla). (D) A worker of *Messor capitatus* transported a shell of Geomitridae, (E) three workers of *Tapinoma nigerrimum* group feed directly on an individual of *Cernuella virgata* (photo credit of both: Carlos del Pico Pons). (F) Two specimens of *Cataglyphis velox* with a *Cernuella virgata* shell (photo credit: Guillermo Albert García). (G) *Messor bouvieri* carrying out a *Theba pisana* shell (photo credit: Miguel Ángel Gómez‐Serrano). (H) *Messor barbarus* carrying a shell of *Cochlicella* sp. (photo credit: Ignacio German Ballesta).

**FIGURE 4:**
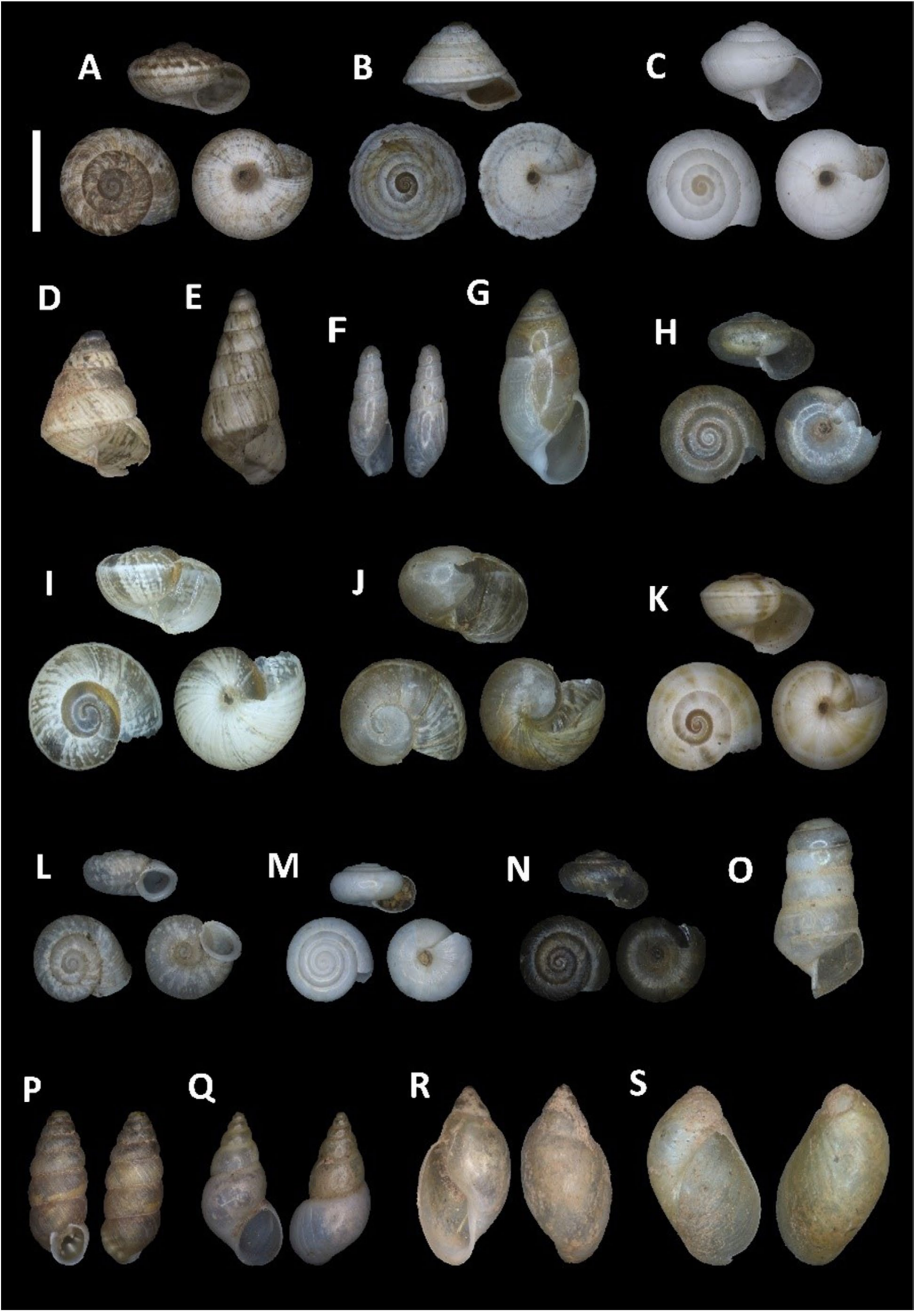
All the snails located in the samplings. Family Geomitridae: (A) *Microxeromagna lowei*, (B) *Trochoidea elegans* , (C) Cernuella virgata*, (D) *Cochlicella barbara* , (E) Cochlicella acuta*. Family Ferussaciidae: (F) *Cecilioides acicula* *, (G) *Ferussacia folliculum*. Family Gastrodontidae: (H) *Zonitoides nitidus* *. Family Helicidae: (I) *Otala* sp.*, (J) Cornu aspersum*, (K) Theba pisana pisana*. Family Valloniidae: (L) *Vallonia costata* . Family Pristilomatidae: (M) *Vitrea contracta* . Family Punctidae: (N) *Paralaoma servilis*. Family Achatinidae: (O) *Rumina decollata* *. Family Chondrinidae: (P) *Granopupa granum*. Family Tateidae: (Q) *Potamopyrgus antipodarum* . Family Physidae: (R) *Physella acuta* . Family Succineidae: (S) Oxyloma elegans*. Juvenile shells are marked with (*). Scale bar 5 mm.

**FIGURE 5:**
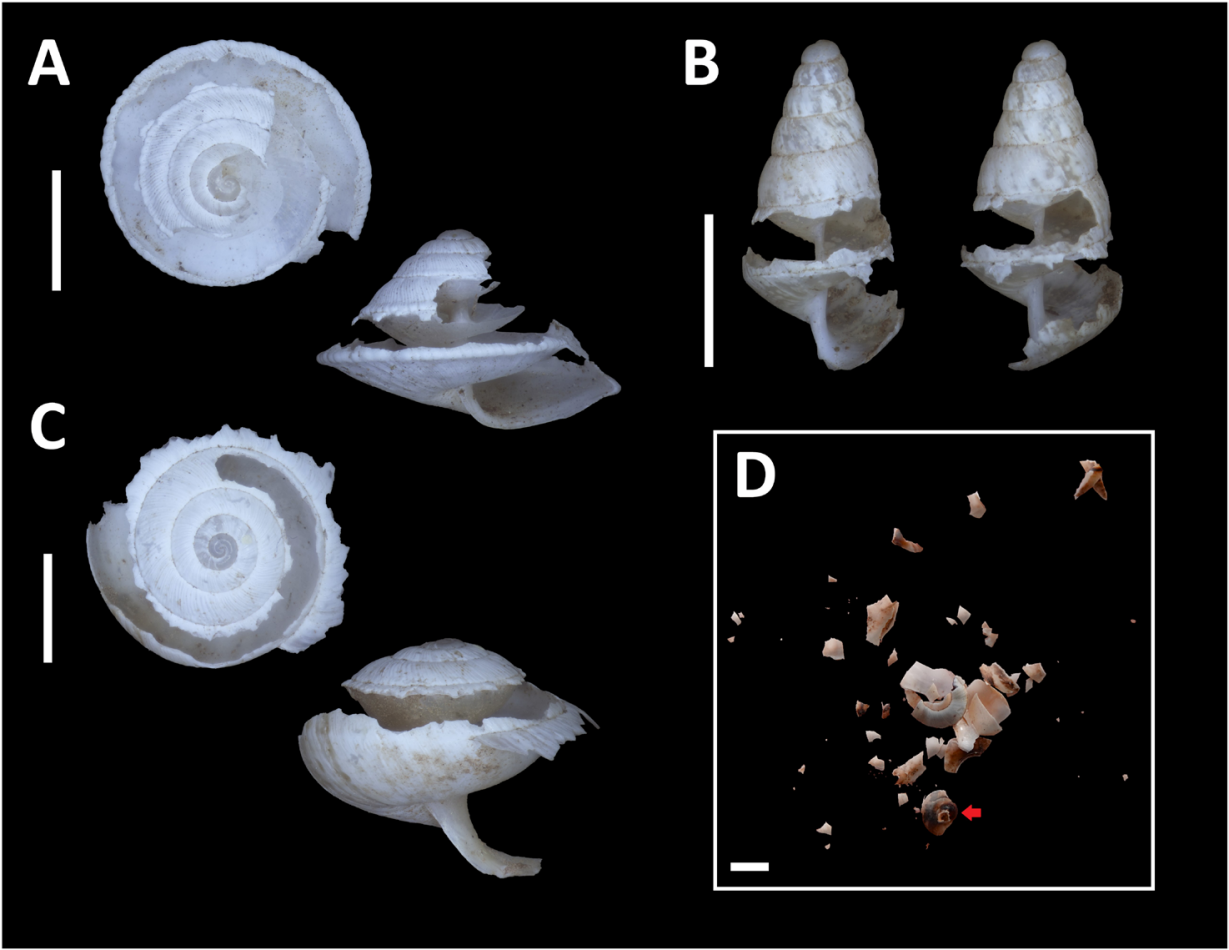
Several shells with holes or spiral perforations located in the ant nest cleanings, concretely (A) *Trochoidea elegans*, (B) *Cochlicella acuta*, (C) *Cernuella virgata*, and (D) the broken shell of *Theba pisana* which a *Messor bouvieri* worker was transporting to its nest after several inspections by ants of the same colony (H44). Noting the reddish mass (snail body remains) attached to one of the pieces of the broken shell.

**FIGURE 6:**
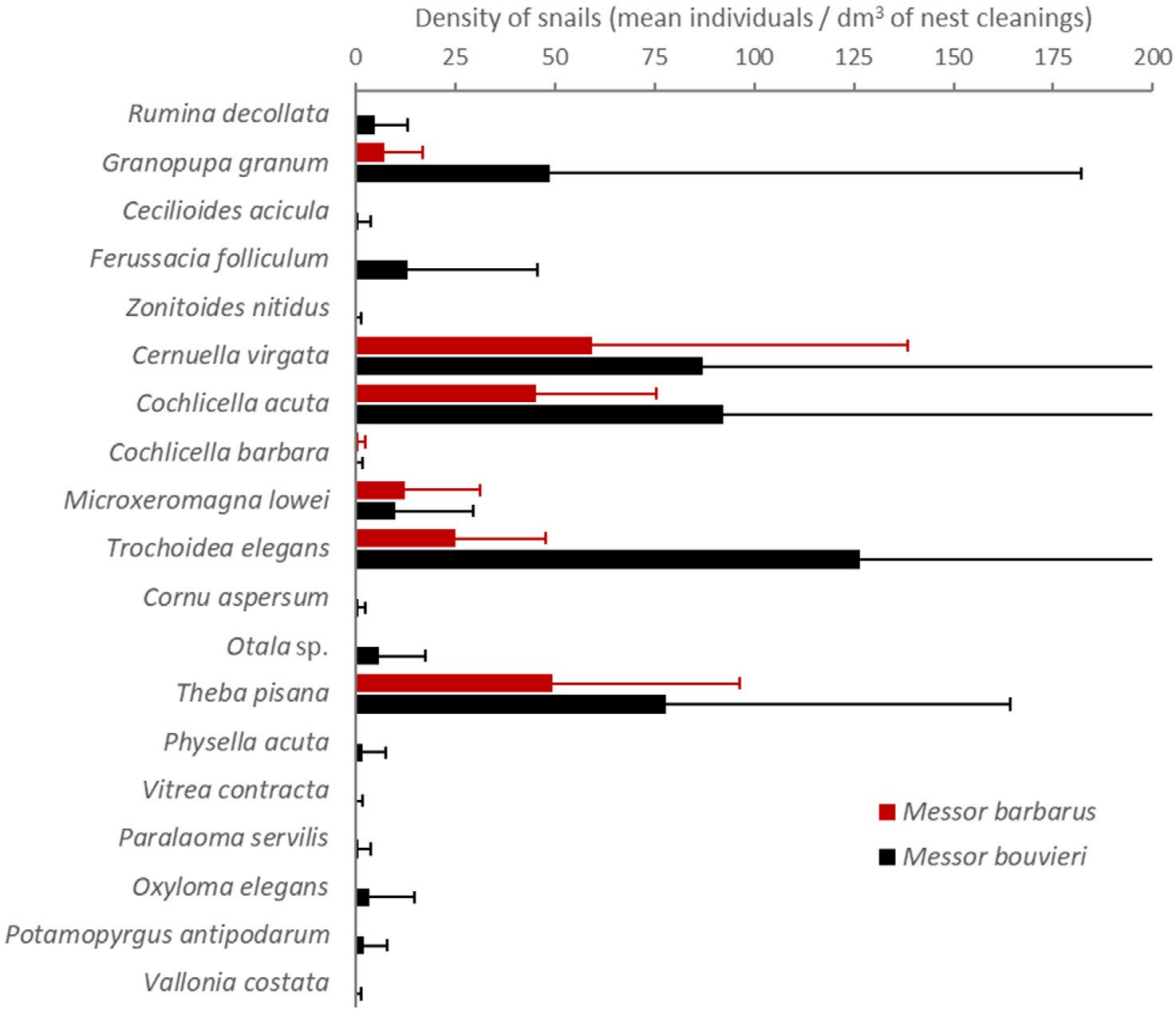
Mean density of the different snail species found in the nest cleanings of the two ant species ( *Messor barbarus* and *M. bouvieri* ). Error bars represent the standard error of the mean.

In total, we have analyzed the cleanings from 4 ant nests of *Messor barbarus* Linnaeus, 1767 and 12 of *Messor bouvieri* (Figure 3A,B). A total of 1146 snail shells from 12 families, 19 genera, and 20 species were collected (Figure 4), of which 168 (14.7%) were found in the nests of *Messor barbarus* (average of 42 shells per nest) and 978 (85.3%) in those of *Messor bouvieri* (average of 80 shells per nest) (Table 1). Most of the shells belonged to snails from the Geomitridae and Helicidae families [756 (66%) and 279 (24.3%), respectively], specifically *Cernuella virgata* (Da Costa, 1778) (26.2% of total), *Theba pisana* (Müller, 1774) (23%), *Trochoidea elegans* (Gmelin, 1791) (19.5%), and *Cochlicella acuta* (Müller, 1774) (17.9%) (Figure 4). The next group was the species *Granopupa granum* (Draparnaud, 1801) (Chondrinidae), of which 70 (6.1%) shells were detected (Figure 4). The remaining families accounted for a total of 41 (3.6%) shells, among which *Ferrussacia folliculum* (Schröter, 1784) (Ferrussaciidae) with 15 (1.3%) and *Rumina decollata* (Linnaeus, 1758) (Achatinidae) with 12 (1.04%) were notable (Figure 4). Of particular interest is the presence of 32 shells (2.8% of the total) with holes or spiral perforations compatible with ant‐mandibles damage (Figure 5A–C). Additionally, although the cleanings were stored after being collected until processing and determination, the species *Granopupa granum* was the unique species with detected five alive individuals in three samples: one in *M. barbarus* nest (H36) and four in *M. bouvieri* nests (3 in H38 and 1 in H49). All detected snail species are terrestrial and native, except for two non‐Iberian‐native and invasive aquatic snail species (Figure 4Q,R): *Physella acuta* (Draparnaud, 1805) and *Potamopyrgus antipodarum* (Gray, 1843) and a native semi‐aquatic one *Oxyloma elegans* (Risso, 1826) (Figure 4S). Regarding the size, it is also important to note that four snail species are considered micromolluscs (< 5 mm) ( *Vallonia costata* (Müller, 1774), *Cecilioides acicula* (Müller, 1774), *Paralaoma servilis* (Shuttleworth, 1852), and *Vitrea contracta* (Westerlund, 1871)), while the others reach larger sizes when adults. The species with the highest mean densities for the whole sample of nests were *Trochoidea elegans* (101.2 ± 241.7 individuals/dm^3^ of nest cleaning), *Cochlicella acuta* (80.4 ± 155.9 individuals/dm^3^), *Cernuella virgata* (80.1 ± 123.7 individuals/dm^3^), and *Theba pisana* (70.7 ± 78.1 individuals/dm^3^) (Appendix 2). In agreement with the fact that there are about twice as many snails per nest in *M. bouvieri* as in *M. barbarus* , the mean densities of the different snail species were higher in *Messor bouvieri* nests than in *Messor barbarus* ones (Figure 6).

**TABLE 1:** Snail shells collected in the sampled ant nests of both *Messor* species. Cases where snails were found alive are marked with (*).

Mollusks/Ants		*Messor barbarus*				*Messor bouvieri*												Total abundance
Family	Species	H14	H35	H36	H42	H26	H28	H3	H38	H39	H43	H44	H49	H52	HG‐2	HG‐3	HX	
Achatinidae	*Rumina decollata*	0	0	0	0	1	0	0	2	0	4	1	0	0	0	0	4	12
Chondrinidae	Granopupa granum*	0	2	2	0	0	1	9	47	1	4	0	3	0	0	0	1	70
Ferussaciidae	*Cecilioides acicula*	0	0	0	0	0	0	0	1	0	0	0	0	0	0	0	0	1
	*Ferussacia folliculum*	0	0	0	0	0	0	0	11	0	1	0	0	0	2	0	1	15
Gastrodontidae	*Zonitoides nitidus*	0	0	0	0	0	0	0	0	0	0	0	0	0	0	0	1	1
Geomitridae	*Cernuella virgata*	0	16	0	50	14	1	0	27	0	61	9	0	14	0	0	108	300
	*Microxeromagna* sp.	1	2	0	0	0	0	0	0	0	0	4	0	0	0	0	1	8
	*Cochlicella acuta*	2	13	1	10	9	5	10	65	8	21	44	1	8	0	0	8	205
	*Cochlicella barbara*	0	0	0	1	1	0	0	0	0	0	0	0	0	0	0	1	3
	*Microxeromagna lowei*	0	0	0	0	2	0	3	7	0	2	0	0	2	0	0	0	16
	*Trochoidea elegans*	0	12	3	5	26	11	7	100	13	13	5	0	14	1	2	12	224
Helicidae	*Cornu aspersum*	0	0	0	0	0	0	0	0	0	1	0	0	0	0	0	1	2
	*Otala* sp.	0	0	0	0	1	0	0	4	0	2	1	0	3	0	0	2	13
	*Theba pisana*	0	22	2	24	22	4	14	23	3	46	58	0	5	0	0	41	264
Physidae	*Physella acuta*	0	0	0	0	0	0	0	0	2	0	0	0	0	0	0	0	2
Pristilomatidae	*Vitrea contracta*	0	0	0	0	0	1	0	0	0	0	0	0	0	0	0	0	1
Punctidae	*Paralaoma servilis*	0	0	0	0	0	0	0	1	0	0	0	0	0	0	0	0	1
Succineidae	*Oxyloma elegans*	0	0	0	0	0	0	0	0	4	0	0	0	0	0	0	0	4
Tateidae	*Potamopyrgus antipodarum*	0	0	0	0	0	1	0	0	2	0	0	0	0	0	0	0	3
Valloniidae	*Vallonia costata*	0	0	0	0	0	0	0	0	0	0	0	0	0	0	0	1	1
Total abundance		3	67	8	90	76	24	43	288	33	155	122	4	46	3	2	182	1146

In the case of presence detection via photos, out of the 67 ant nests, snail shells were detected in 58 nests (86.6%), ranging from 1 to 7 taxa (Table 2 and Appendix 3). The most frequent snail species were *Cochlicella acuta* (50.7% of total nests), *Theba pisana* (46.3%) and *Cernuella virgata* (37.3%) (Appendix 4). From the 9 nests where snails were not detected, five belong to ants of the genus *Messor*: one *M. barbarus* (H15) and four *M. bouvieri* (H34, H39, H49, and H50), and four to non‐*Messor* ants: one *Crematogaster auberti* Emery, 1869 (H21), one *Camponotus sylvaticus* (Olivier, 1792) (H23), one *Cataglyphis iberica* (Emery, 1906) (H32), and one belongs to unidentified species (H24). Additionally, snail shells of the previously mentioned species or families were detected in the ant cleanings of *Aphaenogaster iberica* Emery, 1908, *Camponotus foreli* Emery, 1908, *C. pilicornis* (Roger, 1859), *Cataglyphis iberica*, *Crematogaster auberti* , *Pheidole pallidula* (Nylander, 1849), *Tapinoma nigerrimum* Nylander, 1856, and in eight nests of unidentified ant species different from the previously mentioned. For a more exhaustive view in which ant nests snails were found, see Table 2.

**TABLE 2:** Results of the qualitative assessment of the presence of shells in monitored ant nests of Valencia. The presence of different snails in the ant nest cleanings is marked with (X) when assured and with (?) when impossible to confirm by photographs but with doubts. Abbreviation of the ant species is as follows: Aphibe ( *Aphaenogaster iberica* ), Camfor ( *Camponotus foreli* ), Campil ( *Camponotus pilicornis* ), Camsyl ( *Camponotus sylvaticus* ), Catibe (*Cataglyphis iberica*), Catvel ( *Cataglyphis velox* ) Creaub ( *Crematogaster auberti* ), Mes (*Messor* spp.), Mesbar ( *Messor barbarus* ), Mesbou ( *Messor bouvieri* ), Mescap ( *Messor capitatus* ), Phepal ( *Pheidole pallidula* ), Tapnig (*Tapinoma nigerinum*), and Unid (Unidentified ants). Abbreviations of the snails are as follows: Rumdec ( *Rumina decollata* ), Chond (Chondrinidae family), Ferfol (*Ferrussacia folliculum*), Cervir ( *Cernuella virgata* ), Micr/Xero (*Microxeromagna* or *Xerotricha* indetermined), Xerces (*Xerosecta cespitum*), Cocacu (*Cochlicella acuta*), Cocbar ( *Cochlicella barbara* ), Troele ( *Trochoidea elegans* ), Corasp (*Cornu aspersum*), Otala (*Otala* spp.), Thepis ( *Theba pisana* ), Geo/Heli (Geomitridae or Helicidae unidentified), Phyacu ( *Physella acuta* ), and Oxyele (*Oxyloma elegans*).

Nest code	Ant species	*Rumdec*	*Chond*	*Ferfol*	*Cervir*	*Micr/Xero*	*Xerces*	*Cocacu*	*Cocbar*	*Troele*	*Corasp*	*Otala*	*Thepis*	*Geo/Hel*	*Phyacu*	*Oxyele*	N° of species per nest
H1	Mesbou							X		X			X				3
H1‐1	Creaub				X			X					X				3
H10	Mesbou				X			X					X				3
H11	Mesbou				?												1
H12	Mesbou													X			1
H13	Mesbou									X	X			X			3
H14	Mesbar													X			1
H15	Mesbar																0
H16	Mesbar				X			X									2
H17	Camfor				?												1
H18	Mesbar							X	X					X			3
H19	Mesbar				?			X					X				3
H2	Mes			X	X			X		X	X		X				6
H20	Mesbar							X									1
H21	Creaub																0
H22	Phepal	X			?			X				X					4
H23	Camsyl																0
H24	Unid																0
H25	Camfor													X			1
H26	Mesbou									X			X				2
H27	Mesbou				?	X				X							3
H28	Mesbou				?					X		X	X				4
H29	Creaub												X				1
H3	Mesbou				?			X					X				3
H30	Catibe							X					X				2
H31	Tapnig				X			X		X			X				4
H32	Catibe																0
H33	Mesbar								X					X			2
H34	Mesbou																0
H35	Mesbar									X			X		X		3
H36	Mesbar				?												1
H37	Mesbou							X		X			X				3
H37‐2	Mesbou							X		X			X				3
H37‐3	Mesbou							X		X			X				3
H37‐4	Mesbou							X					X				2
H38	Mesbou				?			X		X			X				4
H39	Mesbou																0
H4	Mesbou							X						X	X		3
H40	Unid							X						X		X	3
H41	Unid				X			X				X	?	X			5
H42	Mesbar							X		X			X	X			4
H43	Mesbou	X						X			X	X	X	X			6
H44	Mesbou				?		?	X					X	X			5
H45	Campil												X				1
H46	Mesbou				?												1
H47	Mesbar							X						X	X		3
H48	Aphibe	X										X					2
H49	Mesbou																0
H5	Mesbou			X		X		X		X			?	X			6
H50	Mesbar																0
H51	Mesbou	X						X					X	X			4
H52	Mesbou							X		X				X			3
H53	Mesbou							X					X				2
H54	Unid													X			1
H6	Mesbou		?					X				X		X			4
H7	Mesbou		?							X		X		X			4
H8	Unid	X			X			X		X		X	?	X			7
H9	Mesbou				?					X			?	X			4
HG1	Mesbou				?			X		X		X	?	X			6
HG2	Mesbou	X		X	?			X				X		X			6
HG3	Mesbou	X		X	X					X			X	X			6
HG4	Unid							X						X			2
HG5	Unid				?									X			2
HG6	Unid													X			1
HG7	Unid			X										X			2
HM1	Mesbar				X				X				X	X			4
HX	Mesbou				X			X					X	X			4
N° of nests with this snail taxon		7	2	5	25	2	1	34	3	20	3	10	31	30	3	1	

### 3.2 Ant–Snail Interactions

The first observation was made on March 8, 2021, in a suburban environment in the city of Seville (southwest Spain; 37.405710, −5.910825). On a small embankment, several ants of the species *M. barbarus* were observed collecting snails in the vicinity of their nest to transport them inside. Although some ants carried empty shells, several workers were reported transporting alive individuals, belonging to the species *Theba pisana* and *Ferrusacia folliculum* (Figure 3C and Video S1). These transported snails were outside their shells, “unprotected”. On several occasions, different individuals of *M. barbarus* were observed approaching to active individuals of *F. folliculum* in the surroundings of their nest, touching them with their antenna but without any attempt of attack or transport. The second observation was made on April 7, 2022, in a Mediterranean scrub in Arteas de Abajo, Castellón (39.902778, −0.745278), where a worker of *Messor capitatus* transported a shell of Geomitridae, to the nest entrance (Figure 3D and Video S2). The third observation, on April 15, 2022, in coastal dunes of El Saler (La Devesa), Valencia (39.3431, −0.309247), three workers of *Tapinoma nigerrimum* (Nylander, 1856) group feed directly on an individual of *Cernuella virgata* after breaking its shell (Figure 3E and Video S3). In the same year, June 17, 2022, in an open field belonging to the Zona de Operaciones Portuarias de Sevilla (southwestern Spain; 37.3327808, −5.9899571) several specimens of *Cataglyphis velox* Santschi, 1929 were observed transporting multiple snails, generally with slightly broken shells, but sometimes complete shells (Figure 3F). Several additional observations were made in Valencia. On October 14, 2022, a worker of *M. bouvieri* was observed transporting an empty shell of a juvenile *Theba pisana* into its nest (nest H26, 39.583570, −0.419061) (Figure 3G and Video S4). Two more observations occurred on October 16, 2022, near one of the *M. bouvieri* nests (H44, 39.583570, −0.419061). First, a worker was observed transporting an empty shell of a juvenile *Cochlicella acuta*, which was eventually taken inside the nest (Video S5). Lastly, several ants were seen exploring a shell of a juvenile *Theba pisana* (Video S6). Notably, while one worker attempted to transport the shell, two others were inspecting it, one from the outside and one from the inside. During the inspection, one ant partially entered the shell while the other went out. Finally, the worker tried to carry out the shell towards its nest (Video S6). We took this shell before the ants introduced it into their nest and broke it in the field. Although the snail was dead, a gelatinous reddish mass of remains of the snail body, was found inside it, attached to the inner part of the shell (Figure 5D). Another observation dates to January 4, 2023, where a *M. bouvieri* ant was seen carrying an empty shell of a juvenile of *Otala* sp. towards one of the entrances of its nest (H28, 39.583134, −0.417934) (Video S7). However, the shell was ultimately left near the entrance and not brought inside due to its size. The last observation dates to April 12, 2024, in El Puntal (Murcia, southeastern Spain; 38.017219, −1.143253). Several *Messor barbarus* workers carried broken or whole shells of live *Cochlicella* sp. individuals into the nest located in an urban garden (Figure 3H and Video S8). A recent case was an observation by A. Baquero and I. Antón (P6) made in a *M. barbarus* nest at the Hermitage of San Babil in Sangüesa (Navarra) between end‐October and first‐November. After several days of rain, workers were observed removing soil from the nest to the outside. On 3 days when the nest was visited (12‐X‐2024, 01‐XI‐2024, and 08‐XI‐2024), 4, 13, and 4 other shells of *C. acicula* (empty shells) were collected, respectively, which the ants had been removing from inside the nest. Additionally, although without exhaustive sampling, throughout the authors' fieldwork for other projects, the presence of living snails in *M. barbarus* nests of at least four snail species has been noted: *Cecilioides acicula* (5 occasions), *G. granum* (3 occasions), *F. folliculum* (several occasions) and, rarely, *Caracollina lenticula* (Férussac, 1821) (2 occasions). In all these cases, snails (adults and juveniles) were found near the nest entrances, or in the nest cleaning area, moving slowly. The ants of the genus *Messor* transported material to the nest among active individuals, without attacking or disturbing them. The snails did not hide in their shells when the ants interacted and remained active even with contact. In most of these cases, snails were seen together with other myrmecophilous taxa such as crickets, terrestrial isopods, beetles or silverfish.

## 4 Discussion

The intriguing relationship between ants and snails has been a scarcely studied topic, with several reported interactions of different nature. The fact that ants transport snail shells to their nest has been reported several times (Butot 1952; Urbański 1965; Mienis 1974; Seidl 1987; Páll‐Gergely and Sólymos 2009; Vaisman and Mienis 2011). Snails are part of the diet of the most specialized ants to the most generalist ones, and there are numerous examples in the literature of this interaction (e.g., Cerdá and Retana 1994; Schatz et al. 1997; Fourcassié and Oliveira 2002; Araújo and Rodrigues 2006; Probst et al. 2019; Carter et al. 2021). Several of our observations (i.e., Videos S1, S3, S6 and S8 and 17th June 2021) confirm several ant species (*Messor barbarus, M. bouvieri, Cataglyphis velox* and *Tapinoma nigerrimun*) preying on several snail species (Figures 3C,E,F,H and 5D). Beyond direct observations, there are other data to make inferences from these cases of predation. We have found 32 shells with perforations compatible with damage from *Messor* mandibles, as already pointed out by other authors (Páll‐Gergely and Sólymos 2009; Bertrand 2010). Harvester ants of the genus *Messor* can incorporate certain species of snails into their diet (Cerdá and Retana 1994; Fernández‐Escudero and Tinaut 1993; Páll‐Gergely and Sólymos 2009; Traxler 2016), which could explain the presence of damaged shells near their nests. Although *Messor* ants are mainly granivorous, they also exhibit scavenging and nectarivorous habits (Arcos and García 2023). Animal tissues apparently often provide key protein foods necessary for animals to develop in their immature stages and reproduce successfully (White 2011). Ants can obtain these animal proteins through scavenging or predation, and the amount and balance of nutrients harvested and the accuracy of regulation depend on the presence of larvae in the colony (Dussutour and Simpson 2009). As an example, it has been reported that primarily granivorous ants, such as Chihuahuan desert seed‐collecting ants (*Pogonomymex rugosus* Emery 1894) regularly eat termites and selectively prey on dense concentrations of grasshoppers (Whitford and Jackson 2007). In certain environments, snails may become easily accessible prey, and their meat could serve as an alternative source of protein, supplementing the diet of harvester ants when other prey are scarce. Snail meat is known to have a high protein content and to provide essential nutrients beyond protein, such as amino acids, fatty acids, vitamins, and minerals, which may not be available in sufficient quantities in a seed‐based diet, making it a valuable nutritional source (Zarai et al. 2011; Çağıltay et al. 2011).

When discussing “hoarding behavior,” we refer to ants collecting small materials near their nest, whether edible or not, for different purposes (Vaisman and Mienis 2011). Some of our observations (i.e., Videos S2, S4, and S5) may refer to this behavior by *Messor* ants. Ants may mistakenly perceive the shells as food resources, like seeds (see Páll‐Gergely and Sólymos 2009; Jahyny 2010; Vaisman and Mienis 2011). Thus, it is quite probable that *Messor* ants may misperceive some shells as other materials, as already mentioned by Vaisman and Mienis (2011). It is important to note that we have detected the presence of snail body remains, forming a reddish mass, in, at least, an empty transported shell. It is possible that ants are collecting empty shells to gather these components as food resources (which might go unnoticed) and then deposit the shell remains in the cleanings. Our observations of *M. bouvieri* interacting with empty shells could be predator behavior or workers evaluating the availability of food remains on the shells which could be taken advantage of. However, considering the significant diversity of species of ants we have reported with shells in their nest cleanings, it is quite improbable that all this species may confuse all shells with other trophic resources. Shell snails are found in high quantities in their nest cleanings (like in Páll‐Gergely and Sólymos 2009; Vaisman and Mienis 2011). This would imply a significant energy cost to transport them, difficult to explain without a specific purpose, as is the case for transporting other types of materials (Smith and Tschinkel 2005, 2007), and probably explanations regarding a food benefit as previously mentioned in the first paragraph need to be considered. However, given the large number of shells usually detected in the nest cleanings, we cannot dismiss other hypotheses for the collection of these non‐edible materials (Smith and Tschinkel 2005). These could be used to modify the nest internal or external temperature, during certain periods, thus increasing the total available activity time for the ant colony (MacKay and MacKay 1985; Smith and Tschinkel 2007). Furthermore, this may serve as direct protection from erosion and perturbation from water (Laundré 1990) and wind (Whitford 2003), to mark their territory or nest with visual cues (Gordon 1984), or may be to reinforce the structure of the nest by filling subterranean gaps. Further studies are needed to assess the functionality of this large number of shells in the nest cleanings, beyond being remains of predation events or used for other purposes.

Regarding myrmecophily, snails are one of the less studied groups. Janssen and Witte (2002) described the first species of snail considered myrmecophilic, based on the interaction behavior between its host ants and the snail (Witte et al. 2002). Additionally, they defined that for a gastropod to be myrmecophilous, it must (a) live within ant nests in the same chambers as immature ants, (b) not suffer aggression when in contact with worker ants, (c) be transported if the colony undergoes relocations, and (d) secrete mucus of a specific nature when interacting with worker ants (Witte et al. 2002). Since then, very few studies have exhaustively reported interactions between ants and snails, probably due to the difficulties in observing them (Heynemann 1868; Verdcourt 2002; Witte et al. 2002; Eguchi et al. 2005; Bertrand 2010; Peixoto et al. 2010; Castaño‐Meneses et al. 2015, 2019; Araujo et al. 2019; Dias‐Soares et al. 2023). We have been able to detect *Messor* ants with a non‐aggressive interaction towards *Ferrussacia folliculum* individuals living in nest entries, as already mentioned by Bertrand (2010). We have also detected that *Granopupa granum, Caracolina lenticula*, and *Cecilioides acicula* are species that were found alive in ant nest entries and cleaning areas, from direct observations and even from the nest cleaning analysis (i.e., *G. granum*). In these cases, snails were always found active and without being attacked by *Messor* workers transporting material to the nest (see Eguchi et al. 2005; Bertrand 2010; Dias‐Soares et al. 2023). So far, snails considered myrmecophilous are found in families such as Achatinidae or Subulinidae, among others (Witte et al. 2002; Eguchi et al. 2005; Dias‐Soares et al. 2023). These families are closely related to the Ferrussacidae as they all belong to the superfamily Achatinoidea (Fontanilla et al. 2017), which could justify the need to study the case of *F. folliculum* as Bertrand (2010) already mentioned, and *C. acicula* , as we comment in this work. However, the case of *G*. *granum* and *C. lenticula* is different as they belong to families far from those with myrmecophilous snails (Wade et al. 2001). Nevertheless, more studies are needed to try to unravel the relationships between these organisms, or even to conclude whether the “myrmecophilous rules” established by Witte et al. (2002) are being followed by several species as it has not completely adhered to in the case of recent observations (Dias‐Soares et al. 2023).

Lastly, it is interesting to highlight that the study of snails in ant nest cleanings also provides other valuable data. Many snail species are difficult to detect due to their small size, often just a few millimeters, or their cryptic habitats in the soil. Studying the diversity of shells in ant nests can give us an approximation of the detection of rare species or an understanding of the malacological fauna of unexplored areas (Páll‐Gergely and Sólymos 2009; Vaisman and Mienis 2011), as is the case with *Cecilioides acicula* , and even invasive species (Vaisman and Mienis 2011) like *Physella acuta* and *Potamopyrgus antipodarum* in our case (Alonso et al. 2019). This is even more interesting, given that these species are aquatic, as the native detected *Oxyloma elegans*. The ant cleanings in which they were found are located around a river, so the presence of these species on the banks is normal, given their amphibious tendencies (Glöer 2019). However, we cannot know whether the ants transported the shells or directly the live specimens, but analyzing ant cleanings is certainly a very useful tool to detect even aquatic (and exotic) species, and not just terrestrial species, as other authors have mentioned (Vaisman and Mienis 2011).

## Author Contributions

**Jairo Robla:** conceptualization (equal), data curation (equal), investigation (equal), methodology (equal), supervision (equal), validation (equal), visualization (equal), writing – original draft (equal), writing – review and editing (equal). **Omar Sánchez:** data curation (equal), formal analysis (equal), writing – review and editing (equal). **Miguel Ángel Gómez‐Serrano:** data curation (equal), formal analysis (equal), investigation (equal), methodology (equal), writing – review and editing (equal). **J. Manuel Vidal‐Cordero:** conceptualization (equal), data curation (equal), investigation (equal), methodology (equal), supervision (equal), validation (equal), visualization (equal), writing – original draft (equal), writing – review and editing (equal).

## Disclosure

Our study was a local approximation based on new data from mostly researchers of different parts of Spain. We tried to look for a collaboration of different scientists not specifically working with the topic of the work but who have observations and want to contribute with their ideas to fill this gap of knowledge.

## Conflicts of Interest

The authors declare no conflicts of interest.

## Supporting information


Data S1.

Video S1.

Video S2.

Video S3.

Video S4.

Video S5.

Video S6.

Video S7.

Video S8.

## Data Availability

All data used for this work is available in the “Results” and in the “Appendix 1, 2, 3, 4” of this work.

## Appendix 1

Data of the location, habitat, and the ant species of the different ant nests and accidental observations included in this work. In the column “Code”, those with “H” refers to ant nest monitored, * to ant nests with analyzed cleanings and “P” to the punctual observations.

**Table:**

Code	Locality	Municipality	Province	Habitat	Ant species
H48	Carraixet	Bétera	Valencia	Dry riverbed	*Aphaenogaster iberica*
H17	Carraixet	Bétera	Valencia	Dry riverbed	*Camponotus foreli*
H25	Carraixet	Bétera	Valencia	Dry riverbed	*Camponotus foreli*
H45	Carraixet	Bétera	Valencia	Dry riverbed	*Camponotus pilicornis*
H23	Carraixet	Bétera	Valencia	Dry riverbed	*Camponotus sylvaticus*
H30	Carraixet	Bétera	Valencia	Dry riverbed	*Cataglyphis iberica*
H32	Carraixet	Bétera	Valencia	Dry riverbed	*Cataglyphis iberica*
H2	Carraixet	Bétera	Valencia	Dry riverbed	*Messor* sp.
H21	Carraixet	Bétera	Valencia	Dry riverbed	*Crematogaster auberti*
H29	Carraixet	Bétera	Valencia	Dry riverbed	*Crematogaster auberti*
H14*	Carraixet	Bétera	Valencia	Dry riverbed	*Messor barbarus*
H15	Carraixet	Bétera	Valencia	Dry riverbed	*Messor barbarus*
H16	Carraixet	Bétera	Valencia	Dry riverbed	*Messor barbarus*
H18	Carraixet	Bétera	Valencia	Dry riverbed	*Messor barbarus*
H19	Carraixet	Bétera	Valencia	Dry riverbed	*Messor barbarus*
H20	Carraixet	Bétera	Valencia	Dry riverbed	*Messor barbarus*
H33	Carraixet	Bétera	Valencia	Dry riverbed	*Messor barbarus*
H35*	Carraixet	Bétera	Valencia	Dry riverbed	*Messor barbarus*
H36*	Carraixet	Bétera	Valencia	Dry riverbed	*Messor barbarus*
H42*	Carraixet	Bétera	Valencia	Dry riverbed	*Messor barbarus*
H47	Carraixet	Bétera	Valencia	Dry riverbed	*Messor barbarus*
H50	Carraixet	Bétera	Valencia	Dry riverbed	*Messor barbarus*
HM1	Carraixet	Moncada	Valencia	Dry riverbed	*Messor barbarus*
H1	Carraixet	Bétera	Valencia	Dry riverbed	*Messor bouvieri*
H10	Carraixet	Bétera	Valencia	Dry riverbed	*Messor bouvieri*
H11	Carraixet	Bétera	Valencia	Dry riverbed	*Messor bouvieri*
H12	Carraixet	Bétera	Valencia	Dry riverbed	*Messor bouvieri*
H13	Carraixet	Bétera	Valencia	Dry riverbed	*Messor bouvieri*
H26*	Carraixet	Bétera	Valencia	Dry riverbed	*Messor bouvieri*
H27	Carraixet	Bétera	Valencia	Dry riverbed	*Messor bouvieri*
H28*	Carraixet	Bétera	Valencia	Dry riverbed	*Messor bouvieri*
H3*	Carraixet	Bétera	Valencia	Dry riverbed	*Messor bouvieri*
H34	Carraixet	Bétera	Valencia	Dry riverbed	*Messor bouvieri*
H37	Carraixet	Bétera	Valencia	Dry riverbed	*Messor bouvieri*
H37‐2	Carraixet	Bétera	Valencia	Dry riverbed	*Messor bouvieri*
H37‐3	Carraixet	Bétera	Valencia	Dry riverbed	*Messor bouvieri*
H37‐4	Carraixet	Bétera	Valencia	Dry riverbed	*Messor bouvieri*
H38*	Carraixet	Bétera	Valencia	Dry riverbed	*Messor bouvieri*
H39	Carraixet	Bétera	Valencia	Dry riverbed	*Messor bouvieri*
H4	Carraixet	Bétera	Valencia	Dry riverbed	*Messor bouvieri*
H43*	Carraixet	Bétera	Valencia	Dry riverbed	*Messor bouvieri*
H44*	Carraixet	Bétera	Valencia	Dry riverbed	*Messor bouvieri*
H46	Carraixet	Bétera	Valencia	Dry riverbed	*Messor bouvieri*
H49*	Carraixet	Bétera	Valencia	Dry riverbed	*Messor bouvieri*
H5	Carraixet	Bétera	Valencia	Dry riverbed	*Messor bouvieri*
H51	Carraixet	Bétera	Valencia	Dry riverbed	*Messor bouvieri*
H52*	Carraixet	Bétera	Valencia	Dry riverbed	*Messor bouvieri*
H53	La Pelosa	Bétera	Valencia	Dry riverbed	*Messor bouvieri*
H6	Carraixet	Bétera	Valencia	Dry riverbed	*Messor bouvieri*
H7	Carraixet	Bétera	Valencia	Dry riverbed	*Messor bouvieri*
H9	Carraixet	Bétera	Valencia	Dry riverbed	*Messor bouvieri*
HG1	Huerto de San Mauro	Godella	Valencia	Scrub and pine forest	*Messor bouvieri*
HG2*	Huerto de San Mauro	Godella	Valencia	Scrub and pine forest	*Messor bouvieri*
HG3*	Huerto de San Mauro	Godella	Valencia	Scrub and pine forest	*Messor bouvieri*
HX*	Carraixet	Bétera	Valencia	Dry riverbed	*Messor bouvieri*
H22	Carraixet	Bétera	Valencia	Dry riverbed	*Pheidole pallidula*
H1‐1	Carraixet	Bétera	Valencia	Dry riverbed	*Crematogaster auberti*
H31	Carraixet	Bétera	Valencia	Dry riverbed	*Tapinoma nigerrinum*
H24	Carraixet	Bétera	Valencia	Dry riverbed	Unidentified ants
H40	Carraixet	Bétera	Valencia	Dry riverbed	Unidentified ants
H41	Carraixet	Bétera	Valencia	Dry riverbed	Unidentified ants
H54	Carraixet	Bétera	Valencia	Dry riverbed	Unidentified ants
H8	Carraixet	Bétera	Valencia	Dry riverbed	Unidentified ants
HG4	Huerto de San Mauro	Godella	Valencia	Scrub and pine forest	Unidentified ants
HG5	Huerto de San Mauro	Godella	Valencia	Scrub and pine forest	Unidentified ants
HG6	Huerto de San Mauro	Godella	Valencia	Scrub and pine forest	Unidentified ants
HG7	Huerto de San Mauro	Godella	Valencia	Scrub and pine forest	Unidentified ants
P1	Sevilla Este	Sevilla	Sevilla	Suburban open area	*Messor barbarus*
P2	Arteas de Abajo	Begís	Castellón	Mediterranean scrub	*Messor capitatus*
P3	El Saler	El Saler	Valencia	Coastal dunes	*Tapinoma nigerrimum* group
P4	Oper. portuarias	Sevilla	Sevilla	Open field	*Cataglyphis velox*
P5	El Puntal	El Puntal	Murcia	Urban garden	*Messor barbarus*
P6	San Babil de Sangüesa	Sangüesa	Navarra	Hole at church wall	*Messor barbarus*

## Appendix 2

Density (Number of individuals/dm^3^ of nest cleanings) of the different snail shells collected in each sampled ant nests of both *Messor* species (corrected from Table 1). Total density is the total amount of snail shells per species found in the total amount of analyzed wastes (dm^3^). Mean density is the mean of densities of each snail shell species in all analyzed 16 nests with their standard deviation (SE density).

**Table:**

Mollusks/Ants		*Messor barbarus*				*Messor bouvieri*												Total density	Mean density	SE density
Family	Species	H14	H35	H36	H42	H26	H28	H3	H38	H39	H43	H44	H49	H52	HG‐2	HG‐3	HX			
Achatinidae	*Rumina decollata*	0	0	0	0	3	0	0	20	0	20	3	0	0	0	0	13	3.9	3.6	7.2
Chondrinidae	*Granopupa granum*	0	9	20	0	0	5	45	470	10	20	0	30	0	0	0	3	23.0	38.3	115.9
Ferussaciidae	*Cecilioides acicula*	0	0	0	0	0	0	0	10	0	0	0	0	0	0	0	0	0.3	0.6	2.5
	*Ferussacia folliculum*	0	0	0	0	0	0	0	110	0	5	0	0	0	40	0	3	4.9	9.9	28.5
Gastrodontidae	*Zonitoides nitidus*	0	0	0	0	0	0	0	0	0	0	0	0	0	0	0	3	0.3	0.2	0.8
Geomitridae	*Cernuella virgata*	0	71	0	167	35	5	0	270	0	305	23	0	47	0	0	360	98.7	80.1	123.7
	*Cochlicella acuta*	80	58	10	33	23	25	50	650	80	105	110	10	27	0	0	27	67.4	80.4	155.9
	*Cochlicella barbara*	0	0	0	3	3	0	0	0	0	0	0	0	0	0	0	3	1.0	0.6	1.2
	*Microxeromagna lowei*	40	9	0	0	5	0	15	70	0	10	10	0	7	0	0	3	7.9	10.6	18.8
	*Trochoidea elegans*	0	53	30	17	65	55	35	1000	130	65	13	0	47	20	50	40	73.7	101.2	241.7
Helicidae	*Cornu aspersum*	0	0	0	0	0	0	0	0	0	5	0	0	0	0	0	3	0.7	0.5	1.5
	*Otala* sp.	0	0	0	0	3	0	0	40	0	10	3	0	10	0	0	7	4.3	4.5	10.1
	*Theba pisana*	0	98	20	80	55	20	70	230	30	230	145	0	17	0	0	137	86.8	70.7	78.1
Physidae	*Physella acuta*	0	0	0	0	0	0	0	0	20	0	0	0	0	0	0	0	0.7	1.3	5.0
Pristilomatidae	*Vitrea contracta*	0	0	0	0	0	5	0	0	0	0	0	0	0	0	0	0	0.3	0.3	1.3
Punctidae	*Paralaoma servilis*	0	0	0	0	0	0	0	10	0	0	0	0	0	0	0	0	0.3	0.6	2.5
Succineidae	*Oxyloma elegans*	0	0	0	0	0	0	0	0	40	0	0	0	0	0	0	0	1.3	2.5	10.0
Tateidae	*Pot. antipodarum*	0	0	0	0	0	5	0	0	20	0	0	0	0	0	0	0	1.0	1.6	5.1
Valloniidae	*Vallonia costata*	0	0	0	0	0	0	0	0	0	0	0	0	0	0	0	3	0.3	0.2	0.8
Total wastes analyzed (dm^3^)		0.3	0.23	0.1	0.3	0.4	0.2	0.2	0.1	0.1	0.2	0.4	0.1	0.3	0.05	0.04	0.3			

## Appendix 3

Relative frequency of the number of snail taxa identified in the nests from the photographs.

## Appendix 4

Percentage of occurrence of the different snail taxa identified in the nests cleanings from the photographs. Abbreviations for snail species: See Appendix 2.

## References

[ece371326-bib-0001] Alonso, Á. , P. Castro‐Díez , A. Saldaña‐López , and B. Gallardo . 2019. “The New Zealand Mud Snail *Potamopyrgus antipodarum* (J. E. Gray, 1853) (Tateidae, Mollusca) in the Iberian Peninsula: Temporal Patterns of Distribution.” BioInvasions Records 8, no. 2: 287–300. 10.3391/bir.2019.8.2.11.

[ece371326-bib-0002] Araújo, A. , and Z. Rodrigues . 2006. “Foraging Behavior of the Queenless Ant *Dinoponera quadriceps* Santschi (Hymenoptera: Formicidae).” Neotropical Entomology 35: 159–164. 10.1590/S1519-566X2006000200002. 17348125

[ece371326-bib-0003] Araujo, E. S. , E. B. A. Koch , J. H. C. Delabie , et al. 2019. “Diversity of Commensals Within Nests of Ants of the Genus Neoponera (Hymenoptera: Formicidae: Ponerinae) in Bahia, Brazil.” Annales de la Societe Entomologique de France 55, no. 4: 291–299. 10.1080/00379271.2019.1629837.

[ece371326-bib-0004] Arcos, J. , and F. García . 2023. “Hormigas de la Península Ibérica e Islas Baleares.”

[ece371326-bib-0005] Bertrand, A. 2010. “Mollusques et Fourmis.” Folia Conchyliologica 5: 3–4.

[ece371326-bib-0006] Bogusch, P. , J. Roháček , P. Baňař , et al. 2019. “The Presence of High Numbers of Empty Shells in Anthropogenic Habitats Is Insufficient to Attract Shell Adopters Among the Insects.” Insect Conservation and Diversity 12, no. 3: 193–205. 10.1111/icad.12335.

[ece371326-bib-0007] Bolton, B. 2024. “AntCat: An Online Catalog of the Ants of the World.” Accessed 29 Jan, 2024. https://antcat.org.

[ece371326-bib-0008] Bondroit, J. 1911. “Contribution à la Faune de Belgique. Notes Diverses.” Annales de la Société Entomologique de Belgique 55: 8–13.

[ece371326-bib-0009] Brown, W. L. , and W. W. Kempf . 1960. “A World Revision of the Ant Tribe Basicerotini.” Studia Entomologica 3: 161–250.

[ece371326-bib-0010] Butot, L. J. M. 1952. “Mieren als Slakkenverzamelaars.” Tropische Natuur 32: 78.

[ece371326-bib-0011] Çağıltay, F. , N. Erkan , D. Tosun , and A. Selçuk . 2011. “Amino Acid, Fatty Acid, Vitamin and Mineral Contents of the Edible Garden Snail (*Helix aspersa*).” Journal of Fisheries Sciences 5, no. 4: 354–363. 10.3153/jfscom.2011040.

[ece371326-bib-0012] Carter, J. , J. Wilson , and S. Mopper . 2021. “Observations of Acrobat Ants (*Crematogaster* sp.) Preying on the Eggs of the Invasive Giant Applesnail (*Pomacea maculata*).” Southeastern Naturalist 20, no. 1: 15–18. 10.1656/058.020.0110.

[ece371326-bib-0013] Castaño‐Meneses, G. , C. S. F. Mariano , P. Rocha , et al. 2015. “The Ant Community and Their Accompanying Arthropods in Cacao Dry Pods: An Unexplored Diverse Habitat.” Dugesiana 22, no. 1: 29–35. 10.32870/dugesiana.v22i1.4173.

[ece371326-bib-0014] Castaño‐Meneses, G. , R. J. Santos , J. R. M. Dos Santos , J. H. C. Delabie , L. L. Lopes , and C. S. F. Mariano . 2019. “Invertebrates Associated to Ponerine Ants Nests in Two Cocoa Farming Systems in the Southeast of the State of Bahia, Brazil.” Tropical Ecology 60, no. 1: 52–61. 10.1007/s42965-019-00006-3.

[ece371326-bib-0015] Cerdá, X. , and J. Retana . 1994. “Food Exploitation Patterns of Two Sympatric Seed‐Harvesting Ants *Messor bouvieri* (Bond.) and *Messor capitatus* (Latr.) (Hym., Formicidae) From Spain.” Journal of Applied Entomology 117, no. 1–5: 268–277. 10.1111/j.1439-0418.1994.tb00735.x.

[ece371326-bib-0017] Dejean, A. , J.‐P. Lachaud , and G. Beugnon . 1993. “Efficiency in the Exploitation of Patchy Environments by the Ponerine Ant Paltothyreus Tarsatus: An Ecological Consequence of the Flexibility of Prey Capture Behavior.” Journal of Ethology 11, no. 1: 43–53. 10.1007/BF02350005.

[ece371326-bib-0018] Del Toro, I. , R. R. Ribbons , and S. L. Pelini . 2012. “The Little Things That Run the World Revisited: A Review of Ant‐Mediated Ecosystem Services and Disservices (Hymenoptera:Formicidae).” Myrmecological News 17: 133–146.

[ece371326-bib-0019] Dias‐Soares, M. , I. M. Correia , J. T. Santos , J. H. C. Delabie , S. D'ávila , and C. S. F. Mariano . 2023. “Facultative Commensalism of Gastropods (Mollusca: Gastropoda) in Neoponera Verenae Forel, 1922 (Formicidae: Ponerinae) Nests.” Insectes Sociaux 1: 5. 10.1007/s00040-024-00956-5.

[ece371326-bib-0020] Donisthorpe, H. S. J. K. 1915. British Ants, Their Life‐History and Classification, 379. Routledge and Sons, Limited.

[ece371326-bib-0021] Dussutour, A. , and S. J. Simpson . 2009. “Communal Nutrition in Ants.” Current Biology 19, no. 9: 740–744. 10.1016/j.cub.2009.03.015. 19345104

[ece371326-bib-0022] Eguchi, K. , T. V. Bui , and R. Janssen . 2005. “Gastropod Guests (Prosobranchia: Pupinidae, and Pulmonata: Subulinidae) Associated With the Ponerine Ant *Diacamma sculpturatum* Complex (Insecta: Hymenoptera: Formicidae).” Sociobiology 45: 307–315.

[ece371326-bib-0023] Emery, C. 1894. “Studi Sulle Formiche della Fauna Neotropica.” Bollettino Della Società Entomologica Italiana 26: 137–241.

[ece371326-bib-0024] Fernández‐Escudero, I. , and A. Tinaut . 1993. “Alimentación no Granívora en *Messor bouvieri* Bondroit, 1918 y *Messor barbarus* (L. 1767) (Hymenoptera: Formicidae).” Boletín de la Asociación Española de Entomología 17, no. 2: 247–254.

[ece371326-bib-0025] Fokuhl, G. , J. Heinze , and P. Poschlod . 2007. “Colony Growth in *Myrmica rubra* With Supplementation of Myrmecochorous Seeds.” Ecologial Restoration 22, no. 5: 845–847. 10.1007/s11284-006-0331-2.

[ece371326-bib-0026] Fonseca, A. M. , and B. S. Sant'Anna . 2020. “Predation on Eggs of the Apple Snail Pomacea Dolioides (Reeve, 1856) in Rural and Urban Areas of the Amazon.” Marine and Freshwater Research 71, no. 6: 662–669. 10.1071/MF19095.

[ece371326-bib-0027] Fontanilla, I. K. , F. Naggs , and C. M. Wade . 2017. “Molecular Phylogeny of the Achatinoidea (Mollusca: Gastropoda).” Molecular Phylogenetics and Evolution 114: 382–385. 10.1016/j.ympev.2017.06.014. 28647619

[ece371326-bib-0028] Forel, A. 1874. “Les Fourmis de la Suisse. Systématique. Notices Anatomiques et Physiologiques. Architecture. Distribution Géographique. Nouvelles Expériences et Observations de Mœurs.” Neue Denkschriften der Allgemeinen Schweizerischen Gesellschaft für di Gesammten Naturwissenschaften 26: 1–452.

[ece371326-bib-0029] Forys, E. A. , A. Quistorff , C. R. Allen , and D. P. Wojcik . 2001. “The Likely Cause of Extinction of the Tree Snail *Orthalicus Reses Reses* (Say).” Journal of Molluscan Studies 67, no. 3: 369–376. 10.1093/mollus/67.3.369.

[ece371326-bib-0030] Fourcassié, V. , and P. S. Oliveira . 2002. “Foraging Ecology of the Giant Amazonian Ant *Dinoponera gigantea* (Hymenoptera, Formicidae, Ponerinae): Activity Schedule, Diet and Spatial Foraging Patterns.” Journal of Natural History 36: 2211–2227. 10.1080/00222930110097149.

[ece371326-bib-0031] Glöer, P. 2019. The Freshwater Gastropods of the West‐Palaearctis. Volume 1: Fresh‐ and Brackish Waters Except Spring and Subterranean Smails. Identification Key, Anatomy, Ecology, Distribution, 399. Svenja Muchow.

[ece371326-bib-0032] Gordon, D. M. 1984. “The Harvester Ant ( *Pogonomyrmex badius* ) Midden: Refuse or Boundary?” Ecological Entomology 9: 403–412. 10.1111/j.1365-2311.1984.tb00837.x.

[ece371326-bib-0033] Headley, A. E. 1943. “The Ants of Ashtabula County, Ohio (Hymenoptera, Formicidae).” Ohio Journal of Science 43: 22–31.

[ece371326-bib-0034] Heynemann, F. D. 1868. “Die Nacktschnecken von der Prinzeninsel—Malakozoologische.” Blätter 15: 32–39.

[ece371326-bib-0035] Hölldobler, B. , and C. L. Kwapich . 2022. The Guests of Ants: How Myrmecophiles Interact With Their Hosts. Harvard University Press.

[ece371326-bib-0036] Jahyny, B. 2010. “Histoire Naturelle du Genre de Fourmis Neotropical *Thaumatomyrmex* Mayr 1887 (Arthropoda, Insecta, Hymenoptera, Formicidae, Ponerinae, Thaumatomyrmecini).” PhD Thesis, Université Paris XIII.

[ece371326-bib-0037] Jahyny, B. , L. S. Ramos‐Lacau , S. Lacau , D. Fresneau , and J. H. C. Delabie . 2003. “A Guilda de Formicidae que Nidificam em Conchas de Gastrópodes Terrestres nos Agrossistemas Cacaueiros do Sudeste da Bahia.” In: Anais do XVI Simpósio de Mirmecologia. UFSC, Florianópolis. pp. 426–428.

[ece371326-bib-0038] Janssen, R. , and V. Witte . 2002. “Allopeas Myrmekophilos n. sp., the First Snail Reported as Living in Army Ant Colonies (Gastropoda: Pulmonata: Subulinidae).” Archiv fur Molluskenkunde 131, no. 1/2: 211–215. 10.1127/arch.moll/131/2002/211.

[ece371326-bib-0039] Kikuchi, T. , and K. Tsuji . 2005. “Unique Social Structure of *Probolomyrmex longinodus* .” Entomological Science 8: 1–3. 10.1111/j.1479-8298.2005.00094.x.

[ece371326-bib-0040] Kistner, D. H. 1982. “The Social Insects' Bestiary.” In Social Insects, edited by H. R. Hermann , vol. 3, 1–244. Academic Press.

[ece371326-bib-0041] Kugler, C. , and M. C. Hincapié . 1983. “Ecology of the Ant *Pogonomyrmex mayri* : Distribution, Abundance, Nest Structure, and Diet.” Biotropica 15: 190–198. 10.2307/2387828.

[ece371326-bib-0042] Laundré, J. W. 1990. “Soil Moisture Patterns Below Mounds of Harvester Ants.” Journal of Range Management 43, no. 1: 10–12. 10.2307/3899111.

[ece371326-bib-0043] Longino, J. T. 1999. “Ants of Costa Rica.” Accessed 20 Dec 2024. http://ants.biology.utah.edu/genera/basiceros/species/manni/manni.html.

[ece371326-bib-0044] MacKay, W. , and E. MacKay . 1985. “Temperature Modifications of the Nest of *Pogonomyrmex montanus* (Hymenoptera: Formicidae).” Southwestern Naturalist 30, no. 2: 307–309. 10.2307/3670749.

[ece371326-bib-0045] Mienis, H. K. 1974. “Mieren als Verzamelaars van Slakkenhuisjes.” Correspondentieblad Van de Nederlandse Malacologische Vereniging 158: 257–258.

[ece371326-bib-0046] MolluscaBase . 2024. Accesed 10 Feb 2024. https://www.molluscabase.org/.

[ece371326-bib-0047] Mukerjee, D. , and S. Ribeiro . 1925. “On a Collection of Ants (Formicidae) From the Andaman Islands.” Records of the Indian Museum 27: 205–209.

[ece371326-bib-0048] Naskrecki, P. , and K. Nishida . 2007. “Novel Trophobiotic Interactions in Lantern Bugs (Insecta: Auchenorrhyncha: Fulgoridae).” Journal of Natural History 41, no. 37–40: 2397–2402. 10.1080/00222930701633570.

[ece371326-bib-0049] Neckheim, C. M. , and P. Boer . 2019. “Slakken in Meirennesten.” Spirulina 421: 27–28.

[ece371326-bib-0050] Oxman, K. , K. M. D. Kuabara , and S. O'Donnell . 2024. “The Terrestrial Isopod *Porcellionides pruinosus* as a Facultative Ant Guest Along Foraging Trails and Inside *Messor ebeninus* Nests in the Negev Desert.” Insectes Sociaux 5: 1–9. 10.1007/s00040-024-01013-x.

[ece371326-bib-0051] Páll‐Gergely, B. , and P. Sólymos . 2009. “Ants as Shell Collectors: Notes on Land Snail Shells Found Around Ant Nests.” Malacologica Bohemoslovaca 8: 14–18. 10.5817/MaB2009-8-14.

[ece371326-bib-0052] Parmentier, T. 2021. “Guests of Social Insects.” In Encyclopedia of Social Insects, edited by C. Starr , 458–472. Springer. 10.1007/978-3-319-90306-4_164-1.

[ece371326-bib-0053] Peixoto, A. V. , S. Campiolo , and J. H. C. Delabie . 2010. “Basic Ecological Information About the Threatened Ant, *Dinoponera lucida* Emery (Hymenoptera: Formicidae: Ponerinae), Aiming Its Effective Long‐Term Conservation.” In Species Diversity and Extinction, edited by G. H. Tepper , 183–213. Nova Science Publishers.

[ece371326-bib-0054] Probst, R. S. 2015. “Revisão Taxonômica e Analise Filogenética de Basiceros Schulz, 1906 (Formicidae, Myrmicinae, Basicerotini).” Master thesis, Museu de Zoologia da Universidade de São Paulo (MZSP), São Paulo, Brazil. 263 pp.

[ece371326-bib-0055] Probst, R. S. , B. D. Wray , C. S. Moreau , and C. R. F. Brandão . 2019. “A Phylogenetic Analysis of the Dirt Ants, Basiceros (Formicidae: Myrmicinae): Inferring Life Histories Through Morphological Convergence.” Insect Systematics and Diversity 3, no. 4: 3. 10.1093/isd/ixz013.

[ece371326-bib-0056] Schatz, B. , J.‐P. Lachaud , and G. Beugnon . 1997. “Graded Recruitment and Hunting Strategies Linked to Prey Weight and Size in the Ponerine Ant *Ectatomma ruidum* .” Behavioral Ecology and Sociobiology 40: 337–349. 10.1007/s002650050350.

[ece371326-bib-0057] Schmid‐Hempel, P. 1998. Parasites in Social Insects. Princeton University Press.

[ece371326-bib-0058] Schultheiss, P. , S. S. Nooten , R. Wang , M. K. L. Wong , F. Brassard , and B. Guénard . 2022. “The Abundance, Biomass, and Distribution of Ants on Earth.” Proceedings of the National Academy of Sciences 119, no. 40: e2201550119. 10.1073/pnas.2201550119. PMC9546634 36122199

[ece371326-bib-0059] Seidl, F. 1987. “Schwarze Gartenameisen (*Lasius niger*) als Schneckensammler.” Mitteilungen der Zoologischen Gesellschaft Braunau 5, no. 1–4: 49–52.

[ece371326-bib-0060] Smith, C. R. , and W. R. Tschinkel . 2005. “Object Depots in the Genus Pogonomyrmex: Exploring the “Who,” What, When, and Where.” Journal of Insect Behavior 18, no. 6: 859–879. 10.1007/s10905-005-8745-1.

[ece371326-bib-0061] Smith, C. R. , and W. R. Tschinkel . 2007. “The Adaptive Nature of Non‐Food Collection for the Florida Harvester Ant, *Pogonomyrmex badius* .” Ecological Entomology 32, no. 1: 105–112. 10.1111/j.1365-2311.2006.00845.x.

[ece371326-bib-0062] Stevens, A. J. , N. M. Stevens , P. C. Darby , and H. F. Percival . 1999. “Observations of Fire Ants (*Solenopsis invicta* Buren) Attacking Apple Snails (*Pomacea paludosa* say) Exposed During Dry Down Conditions.” Journal of Molluscan Studies 65, no. 4: 507–509. 10.1093/mollus/65.4.507.

[ece371326-bib-0063] Stoev, P. , and A. Lapeva‐Gjonova . 2005. “Myriapods From Ant Nests in Bulgaria (Chilopoda, Diplopoda).” Peckiana 4: 131–142.

[ece371326-bib-0064] Tan, H. H. , W. Y. L. Wang , and S. K. Tan . 2019. “Bi‐Coloured Arboreal Ants Apparently Feeding on Eggs of Apple Snail.” Singapore Biodiversity Records 2019: 166–167.

[ece371326-bib-0065] Tennant, L. E. , and S. D. Porter . 1991. “Comparison of Diets of Two Fire Ant Species (Hymenoptera: Formicidae): Solid and Liquid Components.” Journal of Entomological Science 26, no. 4: 450–465. 10.18474/0749-8004-26.4.450.

[ece371326-bib-0066] Tluste, C. , and K. Birkhofer . 2021. “Shells of the Roman Snail Are Important Microhabitats for Soil Invertebrates.” Soil Organisms 93, no. 3: 141–152. 10.25674/so93iss3id167.

[ece371326-bib-0067] Traxler, T. 2016. “Native Food Spectrum, Size‐Matching and Foraging Efficiency of the Mediterranean Harvester Ant *Messor wasmanni* (Hymenoptera: Formicidae).” Ecologica Montenegrina 7: 451–463. 10.37828/em.2016.7.19.

[ece371326-bib-0068] Uchida, S. , H. Mori , T. Kojima , K. Hayama , Y. Sakairi , and S. Chiba . 2016. “Effects of an Invasive Ant on Land Snails in the Ogasawara Islands.” Conservation Biology 30, no. 6: 1330–1337. 10.1111/cobi.12724. 27027403

[ece371326-bib-0069] Urbański, J. 1965. “Ernteameisen als Sammler von Schneckengehäusen.” Mitteilungen der Deutschen Malakozoologischen Gesellschaft 6: 72.

[ece371326-bib-0070] Vaisman, S. , and H. K. Mienis . 2011. “Land Snails in Nest Cleanings of the Black Harvest Ant *Messor ebeninus* in Netzer Sereni, Israel.” Triton 24: 24–28.

[ece371326-bib-0071] Verdcourt, B. 1957. “Snails in Ants' Nests.” Entomologists' Monthly Magazine 93: 41.

[ece371326-bib-0072] Verdcourt, B. 2002. “Two New Species of *Curvella* Chaper (Gastropoda, Pulmonata, Subulinidae) From the East Usambra Mts., Tanzania.” Basteria 66, no. 1–3: 107–112.

[ece371326-bib-0073] Wade, C. M. , P. B. Mordan , and B. Clarke . 2001. “A Phylogeny of the Land Snails (Gastropoda: Pulmonata).” Proceedings of the Royal Society of London. Series B: Biological Sciences 268, no. 1465: 413–422. 10.1098/rspb.2000.1372. PMC1088622 11270439

[ece371326-bib-0074] Wasmann, E. 1894. Kritisches Verzeichniss der Myrmekophilen und Termitophilen Arthropoden. Mit Angabe der Lebensweise und mit Beschreibung Neuer Arten. Verlag von Felix L. Dames.

[ece371326-bib-0075] Weber, N. A. 1941. “The Biology of the Fungus‐Growing Ants. Part 7. The Barro Colorado Island, Canal Zone, Species.” Revista de Entomologia (Rio de Janeiro) 12: 93–130.

[ece371326-bib-0076] Weber, N. A. 1969. “A Comparative Study of the Nests, Gardens and Fungi of the Fungus Growing Ants, Attini.” Proceedings of the 6th Congress IUSSI, Bern 6: 299–307.

[ece371326-bib-0077] White, T. C. 2011. “The Significance of Unripe Seeds and Animal Tissues in the Protein Nutrition of Herbivores.” Biological Reviews 86, no. 1: 217–224. 10.1111/j.1469-185X.2010.00143.x. 20518759

[ece371326-bib-0078] Whitford, W. G. 2003. “The Functional Significance of Cemented Nest Caps of the Harvester Ant, *Pogonomyrmex maricopa* .” Journal of Arid Environments 53: 281–284. 10.1006/jare.2002.1039.

[ece371326-bib-0079] Whitford, W. G. , and E. Jackson . 2007. “Seed Harvester Ants ( *Pogonomyrmex rugosus* ) as “Pulse” Predators.” Journal of Arid Environments 70, no. 3: 549–552. 10.1016/j.jaridenv.2007.01.005.

[ece371326-bib-0080] Witte, V. , R. Janssen , A. Eppenstein , and U. Maschwitz . 2002. “Allopeas Myrmekophilos (Gastropoda, Pulmonata), the First Myrmecophilous Mollusc Living in Colonies of the Ponerine Army Ant Leptogenys Distinguenda (Formicidae, Ponerinae).” Insectes Sociaux 49: 301–305. 10.1007/PL00012646.

[ece371326-bib-0081] Yusa, Y. 2001. “Predation on Eggs of the Apple Snail *Pomacea canaliculata* (Gastropoda: Ampullariidae) by the Fire Ant *Solenopsis geminata* .” Journal of Molluscan Studies 67, no. 3: 275–279. 10.1093/mollus/67.3.275.

[ece371326-bib-0082] Zarai, Z. , F. Frikha , R. Balti , N. Miled , Y. Gargouri , and H. Mejdoub . 2011. “Nutrient Composition of the Marine Snail (Hexaplex Trunculus) From the Tunisian Mediterranean Coasts.” Journal of the Science of Food and Agriculture 91, no. 7: 1265–1270. 10.1002/jsfa.4309. 21328367

