# Supplementary material for: Exploring Ant‐Mollusk Interactions: Insights From Southern Spain
Source: Ecol Evol. 2025 May 7;15(5):e71326. doi: 10.1002/ece3.71326 (PMC12058647; doi:10.1002/ece3.71326)
Supplement: Supplementary file 1 — Data S1. [file ECE3-15-e71326-s007.docx]

We are providing you with a link to download the videos that accompany the research paper due to the upload limit of the Journal of Animal Ecology website:

<https://acortar.link/WqOjex>

Kind regards.
